# The Impact of Biomaterial Surface Properties on Engineering Neural Tissue for Spinal Cord Regeneration

**DOI:** 10.3390/ijms241713642

**Published:** 2023-09-04

**Authors:** Victor A. da Silva, Bianca C. Bobotis, Felipe F. Correia, Théo H. Lima-Vasconcellos, Gabrielly M. D. Chiarantin, Laura De La Vega, Christiane B. Lombello, Stephanie M. Willerth, Sônia M. Malmonge, Vera Paschon, Alexandre H. Kihara

**Affiliations:** 1 Laboratório de Neurogenética, Universidade Federal do ABC, Alameda da Universidade s/n, São Bernardo do Campo 09606-070, SP, Brazil; 2 Department of Mechanical Engineering, University of Victoria, Victoria, BC V8W 2Y2, Canada; 3 Centro de Engenharia, Modelagem e Ciências Sociais Aplicadas, Universidade Federal do ABC, São Bernardo do Campo 09606-070, SP, Brazil; 4 Division of Medical Sciences, University of Victoria, Victoria, BC V8W 2Y2, Canada

**Keywords:** biomaterial surface, tissue therapy, spinal cord injury, cell replacement, human induced pluripotent stem cells, hiPSCs

## Abstract

Tissue engineering for spinal cord injury (SCI) remains a complex and challenging task. Biomaterial scaffolds have been suggested as a potential solution for supporting cell survival and differentiation at the injury site. However, different biomaterials display multiple properties that significantly impact neural tissue at a cellular level. Here, we evaluated the behavior of different cell lines seeded on chitosan (CHI), poly (ε-caprolactone) (PCL), and poly (L-lactic acid) (PLLA) scaffolds. We demonstrated that the surface properties of a material play a crucial role in cell morphology and differentiation. While the direct contact of a polymer with the cells did not cause cytotoxicity or inhibit the spread of neural progenitor cells derived from neurospheres (NPCdn), neonatal rat spinal cord cells (SCC) and NPCdn only attached and matured on PCL and PLLA surfaces. Scanning electron microscopy and computational analysis suggested that cells attached to the material’s surface emerged into distinct morphological populations. Flow cytometry revealed a higher differentiation of neural progenitor cells derived from human induced pluripotent stem cells (hiPSC-NPC) into glial cells on all biomaterials. Immunofluorescence assays demonstrated that PCL and PLLA guided neuronal differentiation and network development in SCC. Our data emphasize the importance of selecting appropriate biomaterials for tissue engineering in SCI treatment.

## 1. Introduction

The spinal cord is a vital part of the central nervous system (CNS) responsible for transmitting signals between the brain and the rest of the body. However, severe damage to the spinal cord can lead to spinal cord injury (SCI), a debilitating condition with complex cellular interactions that often hinders tissue regenerative capacity [1]. The leading causes of SCI include traffic accidents, violence, and falls, which consistently impose significant financial burdens on patients’ families and governmental institutions [2,3]. SCI triggers various clinical symptoms and cognitive impairments, such as loss of sensation and movement, resulting from autonomic response deficits [4]. The mechanical damage that first initiated the SCI is known as the “primary trauma”, which is consequently followed by the “secondary injury”, which comprises the activation of many pathophysiological mechanisms, including the formation of free radicals, delayed calcium influx, exacerbated immune responses, inflammation, and spread of apoptosis throughout the affected area [5,6,7]. Secondary injuries often have more detrimental effects than primary trauma. This is attributed to the extended period of cell death and the initiation of multiple inflammatory responses. Secondary injury intensifies tissue damage, disrupting cellular processes and leading to an enhanced release of inflammatory molecules [8]. As a consequence, these events trigger an intensified inflammatory response, hindering the healing process [9,10]. In the CNS, inhibitory scar tissue formation at the injury site impedes the growth of new neurons and axons, making regenerative treatments challenging. Additionally, the limited regenerative capacity of neurons and inefficient signaling mechanisms within the CNS further hinders self-repair after damage. As a result, current medical interventions for spinal cord injury (SCI) primarily focus on reducing pain, mobility, and sensation in affected limbs through surgeries to relieve nerve pressure and stabilize wounds, complemented by physical therapy [11].

As a strategy to enhance the CNS’s regenerative ability, the transplantation of stem cells at the injury site has shown encouraging results in SCI in rodent models and clinical trials [12,13]. These remarkable cells possess the inherent ability to differentiate into various cell types, including neurons, thus positioning them as potential candidates for repopulating the neuronal environment and facilitating tissue repair [14,15,16]. Such approaches can ensure the replacement of damaged neural cells, network repair, and neurotrophic factor production, leading to fewer inflammatory cytokines and thus promoting a better prognosis of the lesion [17]. However, it is crucial to address the potential risk of stem cells transforming into undesirable cells, necessitating stringent control measures in clinical trials [18]. Understanding the final fate of transplanted cells and assessing teratogen risks becomes paramount for ensuring safety and efficacy in stem cell therapies for spinal cord injuries [15,19].

Biomaterial scaffolds can be used therapeutically when seeded with stem or progenitor cells. Such scaffolds can guide stem cell differentiation and promote a balanced environment for neuronal growth. They can be engineered to stimulate cellular interaction, contributing to the formation of three-dimensional tissues, and mimicking the structure of the original tissue. They could maintain the local and transplanted cells in a stable cellular state by supporting the attachment of autologous cells and placing them at the injury site [20,21].

The extracellular matrix (ECM) is a complex network of proteins and molecules that offer structural support and signaling cues to cells in tissues, playing a critical role in tissue repair and regeneration. To support the incorporation of seeded cells into the local tissue, the biomaterial used in therapies must be biodegradable, helping to restore the natural ECM. As its molecules break down over time, it facilitates cell infiltration and tissue remodeling [22]. In addition, surface properties play a crucial role in determining how cells interact with biomaterials in tissue engineering. Key surface characteristics include roughness, wettability, functional groups, and topography [23]. Roughness influences cell adhesion, proliferation, and differentiation, with controlled roughness promoting neurite outgrowth. Optimizing wettability enhances biocompatibility through improved protein adsorption, cellular response, and tissue integration. Functional groups on the surface regulate cell behavior and guide neurite extension. Finally, surface topography, such as micro- and nanoscale features, can facilitate cell orientation and neurite alignment, contributing to the formation of functional neural networks. The scaffolds can be fabricated from natural or synthetic polymers, although each has exclusive characteristics that should be studied before application [24].

Natural biomaterials exhibit compatible physical, mechanical, and biological properties, along with appropriate degradation rates for cellular events. Moreover, they contain specific signals, such as cell adhesion ligands, that promote effective cell adhesion [25]. Among these natural materials, the polysaccharide chitosan (CHI) can be obtained by alkaline deacetylation of chitin, a resource easily found in shellfish and crustaceans [25]. In addition to its biocompatibility as a natural polymer, the abundance and affordability of CHI derived from marine resources offer significant advantages. Notably, it possesses antibacterial and antitumoral properties while aiding in the wound-healing process [26,27,28].

Indeed, CHI has demonstrated its capacity to effectively promote nerve regeneration in experimental animal models. However, the specific mechanisms by which CHI exerts this regenerative action are not yet fully understood [29]. However, similar to most natural polymers, CHI components can have issues with irreproducibility, instability, and changes during extraction, synthesis, sterilization, or storage, which could affect their biological properties, resulting in sensitive and detrimental formulation characteristics [30].

By contrast, biomaterials from synthetic origins allow better control of the desired characteristics, such as porosity, architecture, stiffness, and degradation rate [31]. Some of the most promising synthetic biomaterials are poly (L-lactic acid) (PLLA) and poly (ε-caprolactone) (PCL) [32]. PLLA is a thermoplastic product from starch, which is found in renewable resources such as potatoes, corn, and sugarcane [33].

PLLA-based polymers possess promising biomaterial potential owing to their essential and versatile properties, including processability, biocompatibility, robust mechanical strength, and biodegradability. Their degradation through nonenzymatic hydrolysis yields lactic acid, a natural product of glucose metabolism in the body, which is safely metabolized through normal physiological pathways [34,35]. In turn, PCL, an aliphatic polyester created from repeated units of hexanoate [36], exhibits low melting temperature, viscoelastic, and semicrystalline properties, allowing the preparation of various scaffold shapes and sizes [37].

In the context of SCI, the combination of stem and progenitor cell transplantation with biomaterial scaffolds shows promising potential as an alternative treatment. This approach can recover lost local cells through the correct differentiation of progenitor and stem cells while providing essential support for neural network formation and guiding axon growth [38,39,40]. However, the interaction between these biomaterials and the local and transplanted cells should be ensured [41]. The scaffold surface warrants primary consideration, as it plays a key role in transmitting signals to the cells. The presence of structures such as native ECM peptide sequences and sulfate proteoglycans on the scaffold surface can influence cellular behavior, leading to substrate remodeling [42,43]. The process of cell adhesion to the biomaterial scaffold can be divided into three stages: (1) cell body attachment to the substrate; (2) cell body flattening and spreading; and (3) cell skeletal reorganization [44]. Moreover, how the cell adheres to the surface of a material can lead to specific transduction of signals in the cells, allowing specific adaptations of their morphology, cytoskeletal dynamics, growth regulation, and even differential gene expression [45]. Consequently, different cell types can display differential responses when attached to the same biomaterial’s surface [46]. Neurons, for instance, as highly differentiated cells that are sensitive to changes in their surroundings, have been described as being very susceptible to these properties [47]. Hence, even though CHI, PCL, and PLLA have been investigated in prior spinal cord studies, identifying the ideal biomaterial for effectively promoting spinal cord regeneration remains uncertain [38,48,49,50,51,52]. It is also essential to recognize and comprehend how the distinctive characteristics inherent to each of these materials might influence cells at the surface level.

Given the polymer diversity and their different surface properties, here we evaluated and characterized the interaction of fibroblast-like cell lines (Vero), spinal cord cells (SCC), neural progenitor cells derived from the neurosphere (NPCdn), and neural progenitor cells derived from human induced pluripotent stem cells (hiPSC-NPC) with natural (CHI) and synthetic biomaterial scaffolds (PCL and PLLA), as well as their impacts on cell proliferation, neural differentiation, and morphology. This process allowed for the identification of biomaterials that could support spinal cord regeneration and have the potential for host stem cell implantation. Taken together, our results represent a fundamental step in understanding how these scaffolds affect cell behavior when viewing tissue engineering for treating SCI.

## 2. Results

### 2.1. The Surface of CHI, PCL, and PLLA Show Distinct Characteristics

**Figure 1:**
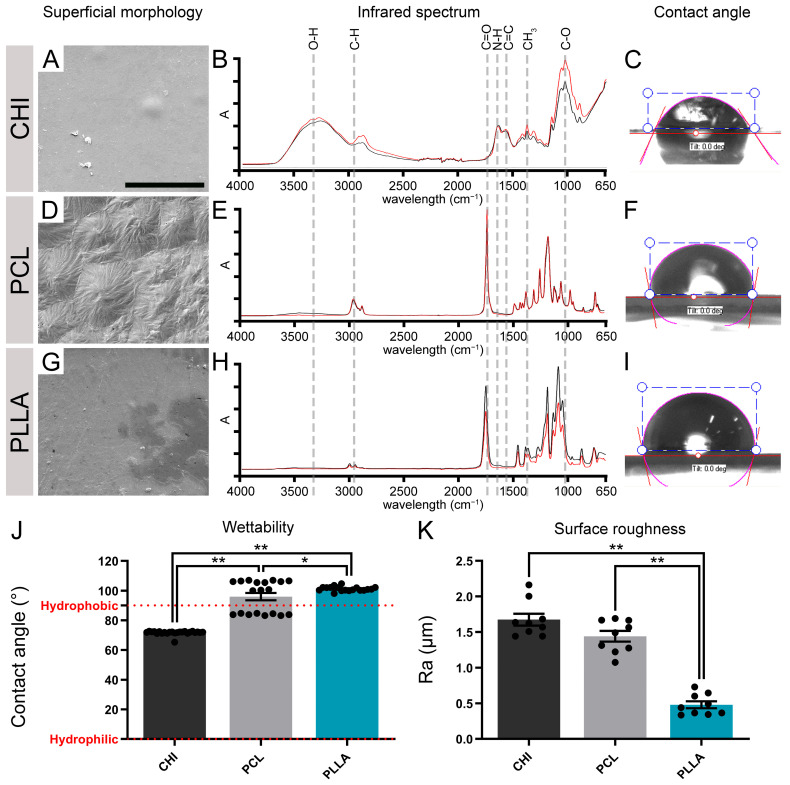
Characterization of biomaterials’ surface morphology, chemical properties, roughness, and wettability. (**A**) Chitosan scaffold (CHI) surface visualized by scanning electron microscopy (SEM). (**B**) Fourier-transform infrared spectroscopy (FTIR) spectrum showing peaks of absorbance that represent functional groups of the CHI surface (O-H, C-H, N-H, C=C, and C-O). (**C**) Representative image of a drop of water on the surface of CHI. (**D**) Poly (ε-caprolactone) scaffold (PCL) surface visualized by SEM. (**E**) FTIR spectrum showing peaks of absorbance (**A**) that represents functional groups of the PCL surface (C-H, C=O, and CH3). (**F**) Representative image of a drop of water on the surface of PCL. (**G**) Poly (L-lactic acid) scaffold (PLLA) surface visualized by SEM. (**H**) FTIR spectrum showing peaks of absorbance (**A**) that represent functional groups of the PLLA surface (C-H, C=O, and CH3). (**I**) Representative image of a drop of water on the surface of PLLA. (**J**) Graph representing the wettability properties of the biomaterials. (**K**) Graph representing the surface roughness (Ra) of the biomaterials. Bars represent standard errors of the mean. Black dots represent the values of each sample. * *p* < 0.05; ** *p* < 0.01. Scale bar: 25 μm.

We performed a physical-chemical analysis to identify the surface characteristics of the tested scaffolds before placing cells in contact with the different biomaterials. Figure 1A,D,G show electron micrographs of CHI, PCL, and PLLA scaffolds showing their surface features. CHI and PLLA showed a smooth aspect, while PCL presented small reliefs (Figure 1A,D,G).

We also analyzed the ATR-FTIR spectra of CHI, PCL, and PLLA scaffolds (Figure 1B,E,H). The infrared spectrum of biomaterials was qualitatively compared, thus evidencing functional groups at the surface. The CHI scaffold presented a broad peak of approximately 3600–3000 cm^–1^ which was associated with O-H, free, and bound hydroxyl groups. The doublet at 3000–2800 cm^–1^ representing C-H stretches was also present in this polysaccharide. Furthermore, approximately 1640–1550 cm^–1^ was found as N-H, characteristic of amine groups. A range of 1600–1475 cm^–1^ was observed as a C=C, related to alkene groups. Peaks between 1325 and 1550 cm^–1^ are related to residual N-acetylated groups. Lastly, approximately 1030–1060 cm^–1^ was detected as C-O stretching vibration, possibly related to COH, COC, and CH_2_OH groups [53]. PCL and PLLA exhibited a doublet at 3000–2800 cm^–1^ as the main bands representing C-H stretches, possibly related to alkane groups. A singlet was detected at 1810–1710 cm^–1^ and associated with C=O bonds, which characterize a carbonyl group. In addition, the range of 1500–1300 cm^–1^ was verified as a splitting band related to CH_3_, a methylene group; however, these peaks were less intense in PLLA when compared with PCL.

The wettability also influences cell behavior and could be assessed by adding a drop of distilled water at room temperature above the scaffold surface, allowing contact angle quantification. The representative image of the water drops and the angle formed with the surface were shown (Figure 1C,F,I), as well as the measured contact angles (Figure 1J). The contact angle obtained for CHI was 71.66 ± 0.28°, for PCL was 95.95 ± 2.45°, and for PLLA it was 101.24 ± 0.35°. The three tested biomaterials were compared, and all the results showed significant differences (*n* = 12, * *p* < 0.05, ** *p* < 0.01).

The surface roughness of biomaterials can also influence cell growth and differentiation [41]. Figure 1K represents the Ra of each biomaterial surface. The Ra determined for CHI was 1.67 ± 0.08 µm, 1.43 ± 0.07 µm for PCL, and 0.47 ± 0.04 µm for PLLA. The sample comparison showed significant differences (*n* = 12, ** *p* < 0.01) between PLLA and the other biomaterials. The lower Ra values obtained for PLLA revealed that its surface is smoother than the other evaluated biomaterials.

### 2.2. The Polymeric Surfaces Exhibited Biocompatibility and Favored the Attachment of Vero Cells

**Figure 2:**
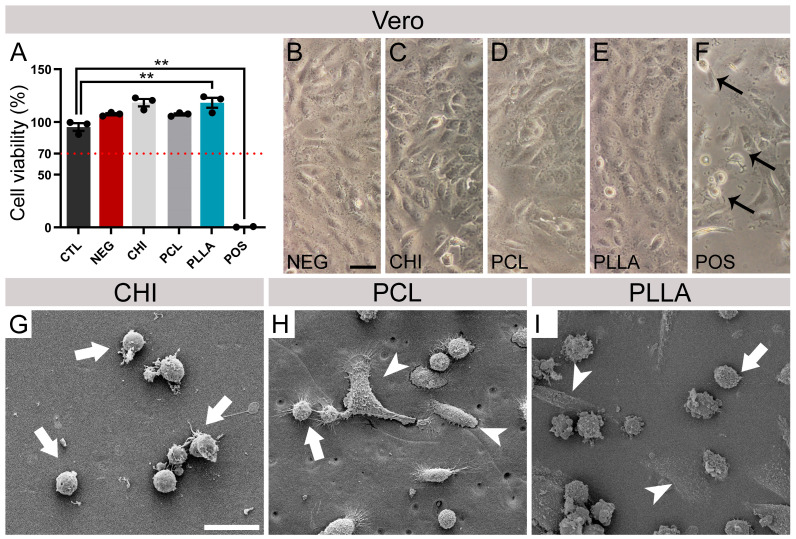
Vero cells viability and adhesion. (**A**) Percentage of Vero cell viability cultivated for 24 h in polymer elution medium in relation to the control (CTL) using the [4,5-dimethyl-thiazol-2-yl] -2,5-diphenyltetrazolium bromide assay (MTT). The dotted red line indicates the 70% threshold of CTL viability, as recommended by ISO 10993-5. Microscope images of Vero cells cultivated in direct contact with (**B**) negative control (NEG), (**C**) chitosan (CHI), (**D**) poly (ε-caprolactone) (PCL), (**E**) poly (L-lactic acid) (PLLA), and (**F**) positive control (POS) with black arrows showing evidence of cellular death. Black arrows indicate signs of cell death. Vero cells were grown on (**G**) CHI, (**H**) PCL, and (**I**) PLLA. White arrows indicate round cells, and white arrowheads indicate flattened cells. Bars represent standard errors of the mean. Black dots represent the value of each sample. ** *p* < 0.01. Scale bar (**B**–**F**): 50 μm. Scale bar (**G**–**I**): 25 μm.

The quantitative MTT assay (Figure 2A) and qualitative direct cytotoxicity morphological evaluation (Figure 2B–F) showed no cytotoxicity response of any tested scaffolds for the Vero cell lineage. According to the data obtained in this assay, after 24 h, all samples were considered noncytotoxic compared to CLT (results above 70% of CTL), for CTL was 100.00 ± 4.28%, for NEG was 95.58 ± 5.02%, 106.85 ± 1.40% for CHI, 106.74 ± 1.39%, for PCL, and 116.98 ± 6.66% for PLLA. POS showed high cytotoxicity, as expected, 6.97 ± 0.32%. Compared to CTL, only PLLA and POS values differed significantly (*n* = 3, *p* < 0.01).

Images obtained using optical microscopy of the NEG showed a confluent monolayer of cells, typically fibroblast-like. After DIV 1, the cells had become scattered without cellular debris or evidence of cell degeneration (Figure 2B–E). Conversely, in the presence of POS, the cells exhibited the typical features of cytotoxicity with cellular debris and cells in suspension, with morphological characteristics of cell death (Figure 2F). The cells cultivated with CHI, PCL, and PLLA scaffolds maintained a pattern of organization in monolayers similar to that observed for NEG, showing a noncytotoxic behavior. Under SEM visualization, it was possible to observe the Vero cells attached to CHI, PCL, and PLLA; however, only round cells were detected on CHI compared with flattened spread cells visualized in PCL and PLLA (Figure 2G–I and Figure S1).

### 2.3. NPCdn Are Sensitive to CHI While SSC Remains Viable in All Materials

**Figure 3:**
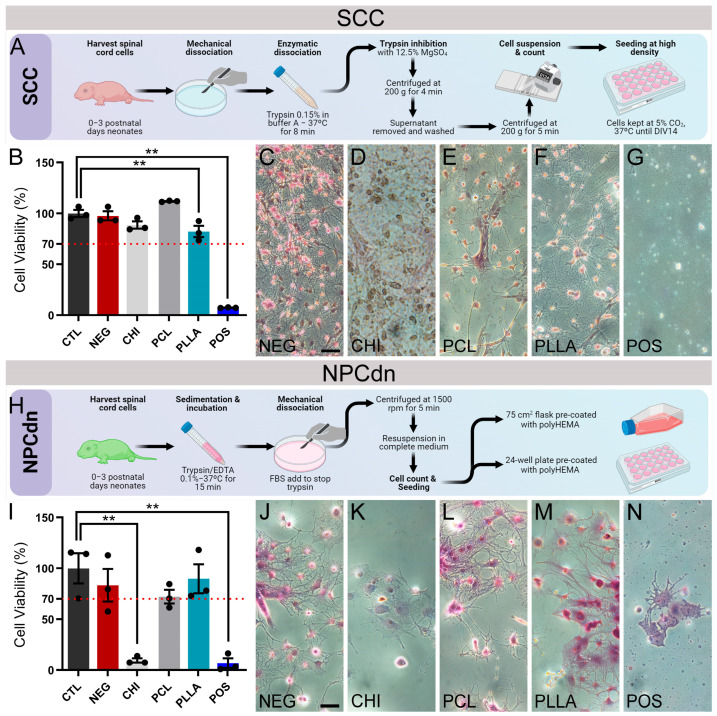
Neuronal and neuro-progenitor cell viability assays. (**A**) Schematic illustration of spinal cord cell (SCC) culture process. (**B**) Percentage of SCC viability cultivated 24 h in polymer elution medium relative to control evaluated by [4,5-dimethyl-thiazol-2-yl] -2,5-diphenyltetrazolium bromide assay (MTT). The dotted red line indicates the 70% threshold of CTL viability, as recommended by ISO 10993-5. Images of cresyl violet stain of SCC cultivated in direct contact with (**C**) negative control (NEG); (**D**) chitosan (CHI), (**E**) poly (ε-caprolactone) (PCL), (**F**) poly (L-lactic acid) (PLLA), and (**G**) positive control (POS). (**H**) Schematic illustration of neural progenitor cells derived from neurospheres (NPCdn) culture process. (**I**) Percentage of NPCdn viability cultivated for 24 h in polymer elution medium relative to control using MTT. Images of cresyl violet stain of NPCdn cultivated in direct contact with (**J**) NEG, (**K**) CHI, (**L**) PCL, (**M**) PLLA, and (**N**) POS. Bars represent the standard error of the mean. Black dots represent the value of each sample. ** *p* < 0.01. Illustration made on Biorender.com. Scale bar: 100 μm.

Quantitative analysis for cell viability of SCC and NPCdn (Figure 3A,H) was performed at DIV 7 using the MTT assay, in which results above 70% of CTL were considered noncytotoxic (Figure 3B,I). In parallel, we performed qualitative tests by direct contact, in which cell morphology was compared among groups (Figure 3C–G,J–N). After 24 h, SCC viability for CTL was 100.00 ± 3.43%, for NEG it was 97.61 ± 4.48%, for CHI it was 88.52 ± 3.60%, for PCL it was 111.81 ± 0.34%, for PLLA it was 82.15 ± 5.52%, and for POS it was 7.32 ± 0.20%. Therefore, all the biomaterials were considered noncytotoxic for SCCs. Statistically significant differences were observed for PLLA (*n* = 3, *p* < 0.05) and POS (*n* = 3, *p* < 0.01) in comparison with the control. Based on these results, all the samples analyzed were considered noncytotoxic since the values in the MTT assay were higher than 70% of CTL (Figure 3B). Under microscopic observation, cells in direct contact with CHI, even with normal mitochondrial metabolism, showed a different morphology and larger and darker cells (Figure 3D). Moreover, PCL and PLLA exhibited cells with a morphologic pattern similar to NEG, branched and bright (Figure 3C,E,F).

However, for the NPCdn, the viability for CTL was 100.00 ± 14.81%, for NEG it was 83.26 ± 16.09%, for CHI it was 9.44 ± 2.14%, for PCL it was 72.10 ± 6.74%, for PLLA it was 89.69 ± 14.24%, and for POS it was 6.86 ± 4.77%. Statistically significant differences were observed for CHI (*n* = 3, *p* < 0.01) and POS (*n* = 3, *p* < 0.01) in comparison with CTL. Then, only CHI was cytotoxic to these cells after 24 h in the extract solution (Figure 3I). In light microscopy, we observed that cells surrounding the CHI sample showed a lower degree of organization than CTL, showing morphological similarities with POS (Figure 3J–N).

### 2.4. CHI Favored the Establishment of Glial Cells, While PCL and PLLA Allowed the Formation of Neuronal Networks

**Figure 4:**
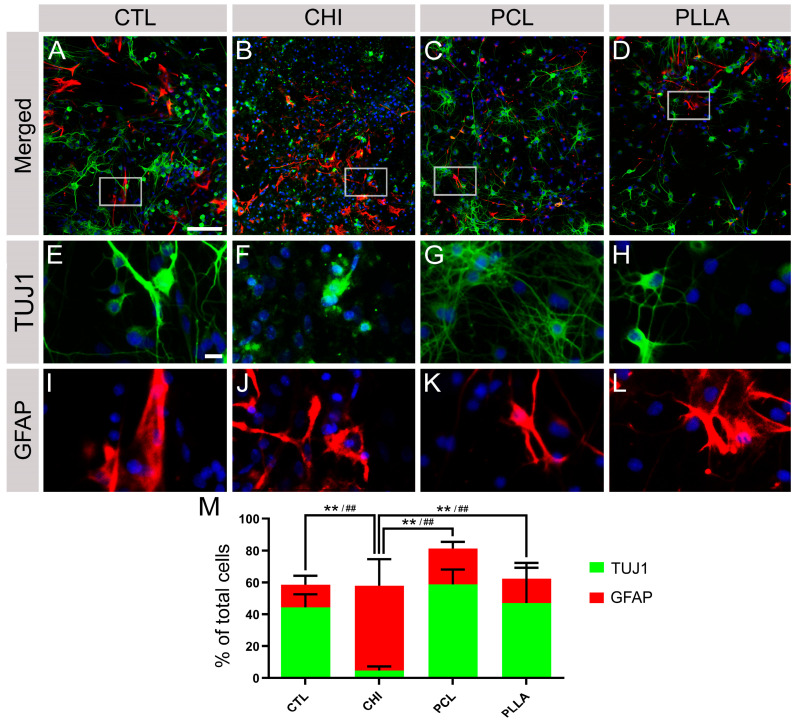
Spinal cord cells (SCC) cultivated in direct biomaterial contact are labeled with anti-βIII-tubulin (TUJ1, green, neurons) and glial fibrillary acidic protein (GFAP, red, astrocytes) and counterstained with 4′,6-diamidino-2-phenylindole (DAPI, blue, cell nucleus). Immunofluorescence of SCC in direct contact with (**A**) control (CTL), (**B**) chitosan (CHI), (**C**) poly (ε-caprolactone) (PCL), and (**D**) poly (L-lactic acid) (PLLA). (**E**–**H**) Zoom boxes with high magnification of the selected area in A–E evidencing TUJ1 labeling. (**I**–**L**) Zoom boxes with high magnification of the selected area in A–E evidencing GFAP labeling. (**M**) Graph representing the percentage of cells labeling TUJ1 and GFAP in each biomaterial. Bars represent standard errors of the mean. ** and ^##^ represent *p* < 0.01 for TUJ1 and GFAP, respectively. Scale bar: 50 μm; scale bar for zoom boxes: 5 μm.

After validating the noncytotoxicity of the biomaterials, SCCs were cultivated in direct contact with the biomaterials to determine the maturation of specific spinal cord cells using fluorescent antibodies raised against neurons (TUJ1) and astrocytes (GFAP) (Figure 4A–L). After DIV 7, SCC in direct contact with biomaterials showed the following proportions of specific cell markers: CTL accumulated TUJ1: 44.23 ± 8.26%, and GFAP: 14.26 ± 5.66%; CHI accumulated TUJ1: 4.64 ± 2.54%, and GFAP: 53.23 ± 16.63%; PCL accumulated TUJ1: 58.72 ± 9.31%, and GFAP: 22.56 ± 4.11%; and PLLA accumulated TUJ1: 47.04 ± 22.17%, and GFAP: 15.23 ± 9.95%. The comparison of each antibody percentage revealed a significant difference (*n* = 5, *p* < 0.01) between CTL and CHI, CHI and PCL, and CHI and PLLA (** and ^##^ represent significant differences for TUJ1 and GFAP, respectively, as shown in Figure 4M). CHI was the only material that did not favor the establishment of neurons; however, astrocytes survived and showed a typical morphology on this material. Indeed, we observed the presence of elongating neurites forming neuronal networks, similar to what is observed in CTL.

### 2.5. NPCdn Spread by the Same Ratio in Direct Contact with Biomaterials; However, CHI Affected the Neurosphere Core Size

**Figure 5:**
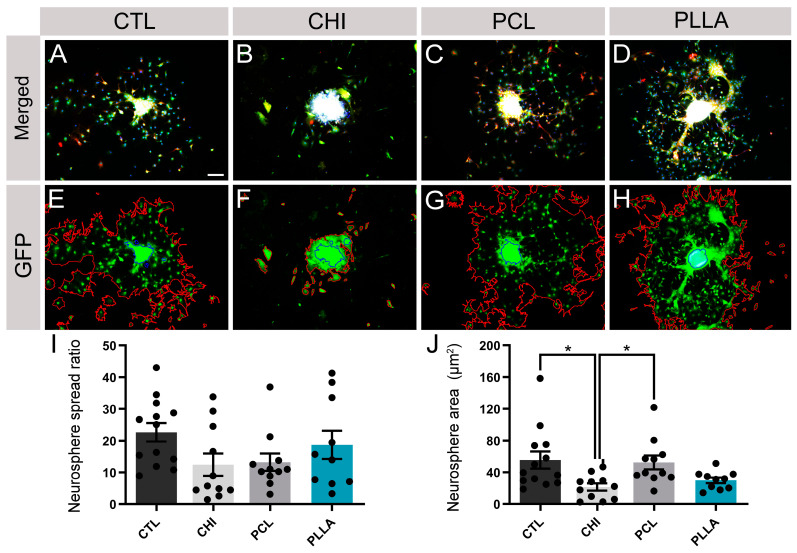
Analysis of the neural progenitor cells derived from neurospheres (NPCdn) spread and core area in the biomaterial scaffolds. NPCdn of green fluorescent protein (GFP)-mouse cultivated in direct contact with the biomaterial scaffolds and labeled with anti-βIII-tubulin (TUJ1, red) and counterstained with 4′,6-diamidino-2-phenylindole (DAPI). Immunocytochemistry of NPCdn-GFP labeling TUJ1 and DAPI: (**A**) control (CTL), (**B**) chitosan (CHI), (**C**) poly(ε-caprolactone) (PCL), (**D**) poly(L-lactic acid) (PLLA). NPCdn-GFP images showing neurosphere and outgrowth area boundaries: (**E**) CTL, (**F**) CHI, (**G**) PCL, and (**H**) PLLA. (**I**) Graph comparing the neurosphere spread ratio. (**J**) Graph comparing neurosphere core area. Bars represent standard errors of the mean. Black dots represent the value of each sample. * *p* < 0.05. Scale bar: 50 μm.

The integration of neuronal progenitor cells into the biomaterial scaffolds is crucial for their survival and spreading [54]. To verify how the tested biomaterials performed in this process, NPCdn-GFP was cultured in direct contact with the proposed scaffolds until DIV 15 (Figure 5A–D). A free software tool called NeuronJ (ImageJ plugin) was used to quantify the cell outgrowth and neurosphere area by obtaining a proportional ratio that expresses the spreading rate of the neurosphere (Figure 5E–H). Thus, the following data were obtained, CTL: 22.66 ± 10.53, CHI: 12.42 ± 11.67, PCL: 13.23 ± 9.05, and PLLA: 18.70 ± 14.10 (unitless values, Figure 5I). The comparison between the samples showed that none of the biomaterials inhibited NPC migration from neurospheres compared to CTL (Figure 5J). Neurosphere core area was also measured, resulting in CTL: 55.53 ± 38.86 μm^2^, CHI: 21.34 ± 15.14 μm^2^, PCL: 52.3856 ± 28.8996 μm^2^, and PLLA: 29.82 ± 11.31 μm^2^, with CHI being the smaller one (*n* = 10, *p* < 0.05).

### 2.6. SCC and NPCdn Were Unable to Adhere in CHI but Attached and Matured in PCL and PLLA

**Figure 6:**
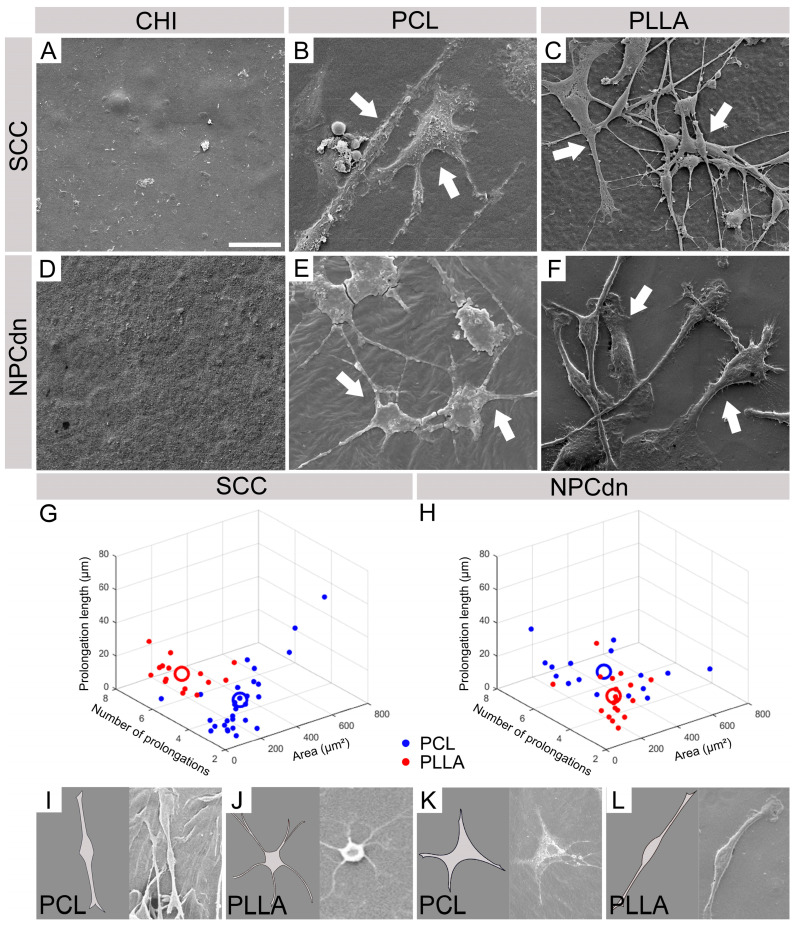
Evaluation of cellular adhesion to the biomaterial surface and acquired morphology. (**A**–**C**) Spinal cord cells (SCCs) grown on (**A**) chitosan scaffold (CHI), (**B**) poly (ε-caprolactone) scaffold (PCL), and (**C**) poly (L-lactic acid) scaffold (PLLA). (**D**–**F**) Neural progenitor cells derived from spinal cord neurospheres (NPCdn) grown on (**D**) CHI, (**E**) PCL, and (**F**) PLLA. White arrows indicate different cells attached to biomaterial surfaces. Scale bar: 25 µm. (**G**) 3D graph representing a cellular population of SCC attached in PCL and PLLA scaffolds. (**H**) 3D graph representing a cellular population of NPCdn attached in PCL and PLLA scaffolds. (**I**–**L**) According to the projection of the centroid in the three axes, values were used for a computational design of a typical cell from each group. Cells exhibiting these characteristics were seen in the analyzed photomicrographs. Representative SCC cultured on (**I**) PCL and (**J**) PLLA scaffolds. Representative NPCdn cultured on (**K**) PCL and (**L**) PLLA scaffolds. Scale bar: 25 μm.

We observed the biomaterial samples under the SEM to verify cellular adhesion and interaction of SCC and NPCdn on the surface of polymeric scaffolds. Neither SCC nor NPCdn were visualized on top of CHI after DIV7 (Figure 6A,D). However, SCC and NPCdn attached and matured in PCL and PLLA (Figure 6B,C,E,F). Moreover, we observed distinct morphologies of SCCs, possibly promoted by the polymer’s interactions (Figure 6B,C), though for NPCdn, this difference was not significant (Figure 6E,F). Cell morphologies were evaluated by randomly selecting 30 cells from each condition, with 10 cells chosen from each of the triplicates representing the distinct PCL and PLLA conditions. The software ImageJ was used to measure the soma area, number of prolongations, and prolongation median length. The obtained data were plotted in a Figure 3D (Figure 6G,H), with X axis: soma area, Y axis: number of prolongations, and Z axis: prolongation median length. A derivate centroid for each condition was then calculated, considering the following values: SCC in PCL X = 150.45 µm^2^; Y = 2.66; Z = 20.82 µm and in PLLA X = 137.87 µm^2^; Y = 5.73; Z = 18.24 µm. NPCdn in PCL X = 276.17 µm^2^; Y = 4.81; Z = 20.30 µm and in PLLA X = 123.68 µm^2^; Y = 2.78; Z = 23.38 µm. According to the projection of the centroid in the three axes, values were used for a computational design of a typical cell from each group. Cells exhibiting these characteristics could be seen inside the analyzed photomicrographs (Figure 6I–L).

### 2.7. hiPSC-NPC Differentiated into Glial Cells after Cultivating on All Biomaterials, While Neurons Spontaneously Emerged in PCL and PLLA

**Figure 7:**
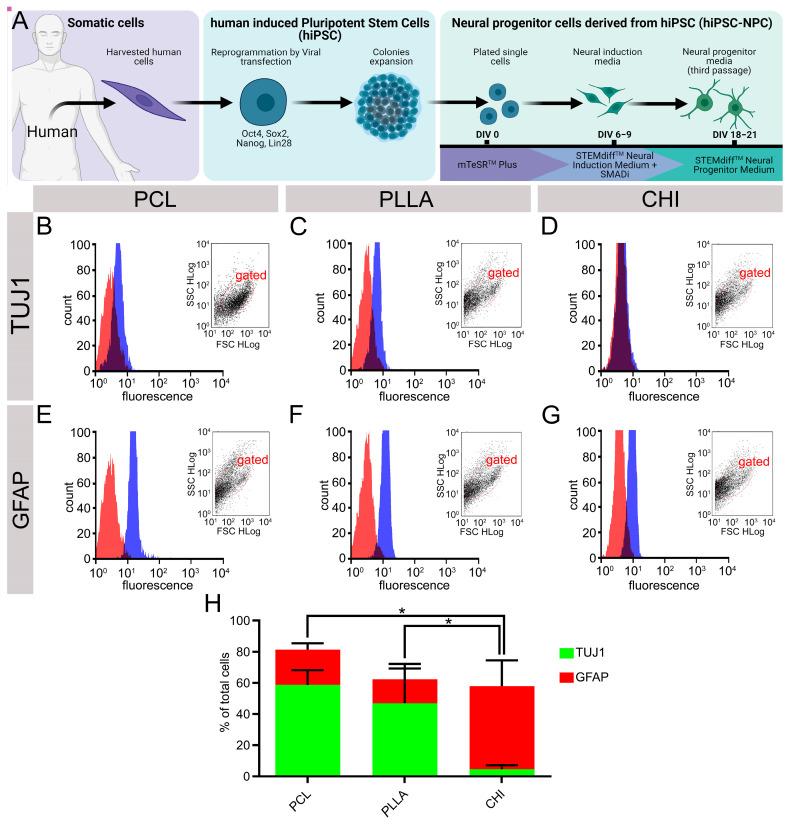
Emergence of βIII-tubulin (TUJ1, neuron) and glial fibrillary acidic protein (GFAP, astrocyte) in neural progenitor cells derived from human induced pluripotent stem cells (hiPSCs-NPC) cultivated over the scaffolds after DIV 15 in free-specific growth factor conditions. (**A**) Schematic illustration of hiPSCs-NPC cultures. **(B**–**D**) TUJ1 marker plotted in flow cytometry graphs of hiPSC-NPC seeded on (**B**) poly (ε-caprolactone) scaffold (PCL), (**C**) poly (L-lactic acid) scaffold (PLLA), and (**D**) chitosan scaffold (CHI). (**E**–**G**) GFAP marker plotted in flow cytometry graphs of hiPSC-NPC seeded on (**E**) PCL, (**F**) PLLA, and (**G**) CHI. (**H**) Percentage of TUJ1 and GFAP in hiPSC-NPC on DIV 15. Bars represent standard errors of the mean. Illustration made on Biorender.com. * *p* < 0.05.

While both SCC and NPCdn demonstrated successful development and attained suitable maturation levels on PCL and PLLA, it is essential to consider the full context of tissue engineering applications. These scenarios often involve the incorporation of autologous stem cells at the site of injury. Therefore, further studies are warranted to explore the preferences and fate of these cells within the scaffolds. After seeding hiPSC-NPC over the biomaterials and incubating them for 15 d, flow cytometry analysis was used to assess the expression of TUJ1 and GFAP markers under conditions devoid of specific growth factors. All the tested scaffolds (CHI, PCL, and PLLA) favored the maturation of glial cells labeled with GFAP over neuronal cells labeled with TUJ1. GFAP expression was the lowest for CHI (52.23 ± 12.16%), increased in PCL (87.82 ± 4.55%), and the highest values were observed for PLLA (94.74 ± 3.15%). The marker TUJ1 was poorly expressed in CHI (1.12 ± 0.35%), increased in PCL (8.87 ± 4.48%), and the highest values were observed for PLLA (13.20 ± 4.87%). Statistically significant differences (*n* = 3, *p* < 0.05) in GFAP expression were observed between CHI and the other samples (Figure 7). Furthermore, no statistically significant differences were observed between the behavior of cells cultured on PCL and PLLA materials and cells cultured in a conventional 2D manner using petri dishes (Figure S2).

## 3. Discussion

In this study, we investigated the impact of distinct surface properties displayed by three prominent polymeric biomaterials-CHI, PCL, and PLLA-on cellular interactions at the surface level. Our approach stands out for its exclusive focus on comprehending how these materials influence cellular behavior. Our exploration of the diverse physical and chemical attributes of biomaterials reveals their profound influence on critical cell behavior and interactions essential for CNS regeneration. These encompass factors such as surface topography, roughness, chemical composition, and wettability. Notably, scaffold surfaces exhibiting the proper hydrophilic-hydrophobic equilibrium and optimal surface topography enhance cell attachment and interaction. Furthermore, the distinct chemical environment provided by each biomaterial, notably influenced by its origin, synthesis and processing, results in varying effects on neuronal and glial development. Moreover, distinct surface attributes actively promote the emergence of diverse CNS cell types and morphological subtypes, highlighting the essential role that biomaterial surfaces play in shaping the behaviors of both locally investigated cells, represented by SCCs, and seeded cells, represented by hiPSC-NPC. Notably, these cells distinctly respond to biomaterial attributes, such as surface topography and chemical composition, yielding diverse cellular morphologies and behaviors. Additionally, the expression of neuronal and glial markers highlights the potential of synthetic polymers PCL and PLLA to encourage neuronal and glial maturation, while the natural biomaterial CHI, under the specific experimental conditions, exhibits inhibitory effects on neuronal development. These collective findings highlight the intricate interplay between biomaterial properties and cellular responses, thereby guiding the design of CNS regeneration strategies. To better grasp these findings, we will delve into each particular feature, exploring them in alignment with the existing literature, in the subsequent paragraphs.

In this context, as we delve into a discussion about the effects of biomaterial surfaces, it becomes crucial to first examine the process of cell adhesion. This dynamic phenomenon is orchestrated by intricate interactions between cell surface molecules and their corresponding ligands in neighboring cells or the extracellular matrix (ECM). Such a process plays a pivotal role in various fundamental aspects of CNS, including maturation, neural network formation, cell communication, regulation of substances, and overall maintenance [42]. The ideal scaffold must provide initial support for cell attachment, proliferation, differentiation, and even secretion of their own ECM [55]. However, we demonstrated that these cell behavioral properties could be influenced by the scaffold surface topography and chemical composition [56], as well as mechanical characteristics [57,58]. In this study, we examined the surface effects of three polymeric biomaterials that are known for their potential in tissue engineering: a natural biomaterial, CHI [59], and two synthetics, PCL [60] and PLLA [61]. These biomaterials have been used for spinal cord regeneration studies [38,48,49,50,51,52], but it is unknown which biomaterial has the more suitable characteristics for SCC and NPCs development. First, we used methodologies to investigate the physical-chemical surface properties. SEM photomicrographs allowed a qualitative observation of small reliefs on CHI and PCL and a smooth aspect for PLLA, characteristics which correlate with the observed Ra in the surface of the scaffold [62,63].

The average roughness can also influence surface wettability or surface energy due to the increase or decrease in the contact area. This parameter describes the adhesive force that favors the solid-liquid interface and provides information about protein adsorption. Consequently, it modulates the cellular shape and adhesion through cytoskeleton reorganization [64]. This significant correlation seems to obey a sigmoidal dependence, which results in an intense transition between excellent and poor cellular adherence [65]. Given the wettability data obtained in this study, CHI was considered significantly more hydrophilic, while PCL and PLLA were considered hydrophobic (high surface energy). It was previously reported that a biomaterial surface needs an appropriate balance of hydrophilic and hydrophobic surface entities, moderate hydrophilicity, and a high surface energy substrate to improve cell adhesion and spreading. These properties promote higher biocompatibility due to the permeation of the culture medium, which allows cells to take advantage of the high surface-area-to-volume ratio offered by the structured substrates. On the other hand, the synthetic polymers provided excellent adhesion, resulting in more spread, flattened cells with surface microvilli, as was previously observed for Vero cells interacting with PLLA [66].

ATR-FTIR revealed different chemical bonds in the tested biomaterials. All the polymers, CHI, PCL, and PLLA, showed patterns according to previous studies [67,68,69,70]. Therefore, superficial molecules associated with low energy bonds (O-H, C-O and others) could be observed in the samples (Figure 1B,E,H).

The Vero lineage is widely used for testing biomaterial cytotoxicity and is regulated by IS0 10993-5:2009 [71,72,73]. None of the tested biomaterials were cytotoxic for Vero cells, as evidenced by indirect and direct tests using the MTT assay to evaluate mitochondrial metabolism and optical microscopy to observe cellular morphology and behavior (Figure 2A–F). This cellular lineage also adhered to CHI, PCL, and PLLA (Figure 2G–I). As visualized by SEM, CHI favored the appearance of rounded cells (Figure 2G), indicating a smaller contact area between the cell’s membrane and the material, possibly influenced by a weaker adhesion. On the other hand, the synthetic polymers provided flattened cells with surface microvilli, as was also previously observed for PLLA [66].

Since neuronal cells are highly differentiated and typically exhibit an elongated morphology and well-branched dendrites, also portraying a low proliferation rate, we repeated the viability tests with SCC and NPCdn to confirm the noncytotoxic effects of the biomaterials for the target tissue. To our surprise, none of the polymers was cytotoxic for SCC (Figure 3A), but CHI was cytotoxic for NPCdn, showing a mitochondrial metabolism of less than 10% compared to CTL (Figure 3I). In the direct contact test, both SCC and NPCdn surrounding CHI exhibited a different morphology pattern as expected by the NEG group, while other polymeric scaffolds showed the presence of cells with typical characteristics (Figure 3C–G,J–N). Previous studies suggest that the concentration of CHI on the film surface could affect the proliferation and viability of postmitotic neurons (NGF-differentiated PC12) [35].

Previous studies have demonstrated that CHI exhibits significant immunostimulatory activity, inducing both pro- and anti-inflammatory environments [74]. Derived from chitin, CHI consists of linear copolymers of D-glucosamine (deacetylated units) and N-acetyl-D-glucosamine (acetylated units) linked by β-(1,4) glycosidic bonds. The deacetylation process determines the degree of deacetylation, influencing the amino content in chitosan chains [75]. The toxicological profiles of CHI are influenced by its degree of deacetylation and molecular weight [76], which directly impact its charge density. A higher degree of deacetylation and molecular weight result in a greater number of positively charged amino groups in chitosan, leading to a higher charge density [77,78]. At high levels, this feature can affect the capability to produce reactive oxygen species, bind to receptors, and impact cellular membranes, leading to particle dispersion and aggregation [79]. Consequently, these factors may be associated with the lower viability of immature neurons and unexpected morphology of SCC and the neural progenitor cells NPCs in direct contact with CHI.

As none of the tested polymers were cytotoxic to SCC, we assessed whether direct contact of these cells with the biomaterials promotes the maturation of specific spinal cord cell types using TUJ1 to label neurons and GFAP to label astrocytes (Figure 4A–L). We observed that CHI negatively affected neuronal maturation but instead allowed the development of astrocytes at a higher ratio (Figure 4M). Similar outcomes were observed in previous CNS reports where CHI nanoparticles as a drug delivery system reduced neuronal viability and neurite extension while impairing neural stem cell migration [80]. This effect could be attributed to the high charge density of CHI, creating a proneuroinflammatory environment that damages neurons but supports glial cells [81]. Thus, while CHI has shown both anti-inflammatory and proregenerative effects in previous studies [29], its properties, influenced by the acetylation pattern and molecular weight, lead literature to divergent findings on its pro- or anti-inflammatory behaviors in CNS [74]. In contrast, PCL and PLLA demonstrated the stimulation of neuronal maturation, with some astrocytes, indicating their potential as promising materials for nerve guide use (Figure 4M).

Since only CHI affected the maturation of SCC, representing the local cells from the spinal cord in the SCI scenario, we evaluated the behavior of NPCdn, a possible option for pluripotent exogenous cells to be delivered through polymers. The behavior of neurosphere-derived NPCs was analyzed by measuring the spreading area, which is a parameter that relates the proportion between the neurosphere core and outgrowth areas. Although no evident differences were detected in the neuronal spread in any of the tested scaffolds (Figure 5I), smaller neurosphere cores and outgrowth were observed on NPCdn seeded on CHI (Figure 5J), which could have been affected by the high charge density stimulus of this biopolymer.

Knowing the effects of direct contact with biomaterials, we seeded the SCC and NPCdn cells on top of the scaffolds to assess their adhesion. Recent studies have highlighted variations in the growth patterns of osteoblasts based on surface hydrophilicity and hydrophobicity. High surface energy (hydrophilic) surfaces have been found to facilitate even and uniform spatial growth of human fetal osteoblastic cells, while low surface energy (hydrophobic) surfaces tend to exhibit random clustering of cells, forming clump-like structures [82]. Similarly, our findings suggest that neural cells can also adhere and mature differently on hydrophilic or hydrophobic surfaces. After DIV 14, no attached cells could be found on CHI (Figure 6A,D), but only in the materials considered hydrophobic, PCL (Figure 6B,E), and PLLA (Figure 6C,F). Among these samples, we observed well-branched cells with different morphologies for SCC, though, for NPCdn, no clear morphology difference was detected. To analyze cell morphology and if there were specific populations for PCL and PLLA, we plotted representative cells in 3D. SCC showed fewer extensions and higher soma areas in the PCL scaffold graph (Figure 6G). When seeded over PLLA, SCC showed a larger branch extension than PCL (Figure 6G). For NPCdn, two populations were observed, similar to SSC (Figure 6H). NPCdn attached to PCL exhibited a higher soma area and more prolongations when compared to PLLA (Figure 6H). Aligned with our results, some studies have also reported that PLA-based scaffolds can stimulate neurite extension [83,84,85].

The impact on cellular adhesion, maturation, and differentiation is not solely confined to hydrophilicity; it also extends to the physical-chemical composition and charge of the scaffold surface, as elaborated earlier [86]. Some studies suggest that even the same material surface can lead to differential responses in several tissues [87]. These features correlate with our experimental observations since different neuronal types and subtypes emerged on the same biomaterial surface.

Even after describing SCC and NPCdn behavior on the scaffolds, it is essential to elucidate how human stem cells would react after being seeded on these polymers. It was previously reported that natural polymers, such as scaffold-based on fibrin [88], and synthetic polymers, such as poly (ethylene glycol) [89], can promote the emergence of neuronal and glial markers from hiPSC-NPC. In this study, hiPSC-NPC was seeded onto the tested biomaterials in the absence of specific growth factor conditions. To ensure a suitable time for differentiation and maturation, the cells were incubated until DIV 15 [90]. In these conditions, CHI showed the lowest levels of TUJ1 and GFAP in comparison to the synthetic polymers (Figure 7H). These results suggest that CHI could not promote an optimal environment for neuronal and glial development, possibly, due to the high charge density of CHI molecules that reduced the emergence of astrocyte and neuronal features from hiPSC-NPC.

On the other hand, PCL and PLLA favored the differentiation of glial and neurons, at lower proportions for the latter. It is important to reinforce that no specific growth factor was used, allowing the higher glial differentiation since it is easy to occur in these environmental conditions and the intrinsic phase of progenitor cells [91,92]. Consistently with our results, it was also reported previously that a PCL-based biomaterial promoted the expression of TUJ1 in hiPSC-NPC [93]. Studies have shown that tuning the immunomodulatory surface of polymeric biomaterials has improved bone tissue integration [94]. Then, to enhance and direct neuronal differentiation, hiPSC-NPC in contact with these scaffolds should be treated with growth factors and small molecules, such as B27, N2, l-ascorbic acid, retinoic acid, and purmorphamine [88,95,96]. Moreover, new technologies based on gene therapy, such as clustered regularly interspaced short palindromic repeats (CRISPR), optogenetics, and designer receptor exclusively activated by designer drugs (DREADDs) can be used to engineer cells, creating conditions to better control the cell fate during the tissue engineering for spinal cord restoration [97].

To gain a comprehensive understanding of their maturation, communication properties, and interaction within distinct biomaterial layers, employing three-dimensional models with neural cells cultured overlayered onto the materials is imperative. These models possess the capability to replicate the intricate effects of cell-to-cell and cell-to-ECM interactions, while also offering an environment that closely mimics in vivo conditions [98]. This advantage enhances the practical application of tissue engineering techniques and deepens our comprehension of cellular responses in a more physiological context. Lastly, the relations found in this study between scaffold surface and neuronal cells can be used as complementary information to predict the behavior of nervous tissue when using other possible biomaterials in the early development of in vivo trials.

## 4. Materials and Methods

### 4.1. Biomaterial Preparation

In this project, the polymeric samples utilized were provided by the Federal University of ABC research group on biomaterials, under the leadership of Prof. Dr. Sonia M. Malmonge. The material preparation was conducted as follows:

A solution of medium molecular weight chitosan (CHI, 448877, Sigma Aldrich, St. Louis, MO, USA) at a concentration of 1% *w*/*v* was first formulated using an aqueous acetic acid solution (3% *w*/*v*) as the solvent, followed by stirring for a duration of 24 h. According to the manufacturer’s specifications, this particular CHI variant possesses a molecular weight falling within the range of 190,000–310,000 Da, coupled with a deacetylation level of at least 75%. The solution was poured into a Petri dish and remained in a fume hood until complete solvent evaporation. The resulting film was immersed in 1M NaOH (Synth, Diadema, SP, Brazil) solution for approximately 40 min to complete acid neutralization. The films were carefully removed, thoroughly washed in distilled water, dried at room temperature, and stored under vacuum (–100 Pa).

PCL Mn 80.000 (440744, Sigma Aldrich, St. Louis, MO, USA) and PLLA (2002D, Nature Works, Minnetonka, MN, USA) films were obtained by solvent evaporation. PCL and PLLA polymers were solubilized in chloroform (CHCl_3_; Synth, Diadema, SP, Brazil) (5% *w*/*v*) and stirred for 24 h at room temperature. The solutions were poured into Petri dishes and kept in a fume hood until complete evaporation of the solvent. The resulting films were stored under vacuum.

### 4.2. Analysis of the Biomaterials’ Surface

Scanning electron microscopy (SEM, Quanta 250TM, FEI Co. ThermoFisher Scientific, Waltham, MA, USA) was used to characterize the surface of polymeric scaffolds. The samples were covered with a thin layer of gold (15 nm) by sputtering (Leica ACE200, Leica Microsystems, Wetzlar, HE, Germany) at room temperature. After metallization, the samples were stored under vacuum until SEM analysis. Images were taken under high vacuum at different magnifications at 5 kV.

### 4.3. Chemical Properties of the Biomaterials’ Surface

Infrared spectroscopy (ATR-FTIR, PerkinElmer Frontier Single & Dual Range spectrometer, PerkinElmer, Waltham, MA, USA) was used to evaluate the surface chemical properties of the biomaterial scaffolds. Each sample had 60 scans with a resolution of 0.4 cm^–1^ corresponding to the 6000–450 cm^–1^ spectra region, following the previously reported method [99].

### 4.4. Roughness

A roughness test was performed to complement the morphological analysis of sample surfaces, thus allowing quantitative evaluation of the sample surfaces’ average roughness (Ra). For this analysis, the membranes were fastened (CHI, PCL, and PLLA) to a flat surface using a glass slide to stretch the material and avoid folds or irregularities. The Ra was measured using the roughness meter TR200 (TIME Group, Beijing, China). During the procedure, the sensor moves linearly along the sample surface. The resulting movements provide the Ra value.

### 4.5. Wettability

To evaluate the hydrophilicity or hydrophobicity properties, we employed the sessile drop assay and measured the contact angle (θ). Water drops were deposited with a syringe on the surface of polymeric scaffolds, which were fixed to microscope slides with double-sided adhesive tape. Contact angles were obtained by leaving the syringe tip in the droplet and adding or siphoning liquid. An Attension contact angle goniometer and Attension theta optical contact angle (Biolin Scientific, Stockholm, VG, Sweden) were used for this analysis. Data were analyzed using the Pendant Drop Surface Tension software (v2.5, Biolin Scientific, Stockholm, VG, Sweden). The samples were classified as superhydrophobic (if the θ was above 150°), hydrophobic (if the θ was between 90° and 150°), or hydrophilic (if the θ was less than 90°) [100].

### 4.6. Scaffold Sterilization

Samples of the tested scaffolds were sterilized by ultraviolet (UV) irradiation for 30 min on each side of the material (total: 1 h) at KW 254 nm LW 365 nm. Samples were placed 9 cm from the UV source inside a laminar flow hood.

### 4.7. Vero Cell Culture

This study used the Vero cell lineage to characterize polymeric biomaterials’ cytotoxic behavior and adhesion characteristics (Adolfo Lutz Institute, São Paulo, SP, Brazil). This fibroblast-like cell line was established from African green monkey kidney epithelial cells (*Cercopithecus aethiops*) [101]. Cells were seeded at a high density, 10 × 10^4^ cells/ml, and cultured in Ham F-10 medium (Sigma Aldrich, St. Louis, MO, USA) containing 10% fetal bovine serum (FBS) at 37 °C with 5% CO_2_.

### 4.8. Spinal Cord Cell (SCC) Culture

For the evaluation of specific tissue response, neonates (males and females) of Wistar rats (Rattus norvegicus) with 03 postnatal days (P0–P3) were used (animal handling protocol approved by CEUA-UFABC 4509160816). After euthanasia by decapitation, the spinal cord segments were gently dissected, and the meninges and vessels were carefully removed. The tissue was gently washed three times with buffer A (5.4 mM of KCl, 1.2 mM of KH_2_PO_4_, 136.9 mM of NaCl, 4.2 mM of sodium bicarbonate NaHCO_3_, 352.2 µM of Na_2_HPO_4_, 5.5 mM of dextrose in distilled water at pH 7.4) containing antibiotic (10 µg/mL of penicillin/streptomycin). The tissue was mechanically dissociated with a scalpel blade in a petri dish, transferred to a 15 mL conical tube, and finally dissociated enzymatically with trypsin 0.15% in buffer A (Trypsin 2.5% 10X, 15090046, ThermoFisher Scientific, Waltham, MA, USA) at 37 °C for 8 min. After this incubation, trypsin action was inhibited by adding 60 µL of a solution of 12.5% MgSO_4_ diluted in distilled water, and the cells were centrifuged at 200 g for 4 min. The supernatant was discarded with a Pasteur pipette and washed with buffer A before centrifuging at 200 g for 5 min, and this procedure was repeated twice. A cell suspension sample (10 µL) was counted in a hemocytometer to determine cell concentration. The cells were plated at a high density of 2.5 × 10^4^ cells/mL with neurobasal plating media (Dulbecco’s Modified Eagle Medium, 10% of FBS, 1% of N-2 Supplement 100X and 10 µg/mL of penicillin/streptomycin) on poly-D-lysine coated dishes and directly on the surface of evaluated scaffolds for adhesion assay. The media was changed to neurobasal growth media (Dulbecco’s Modified Eagle Medium, 10% of FBS, 1% of N-2 supplement 100X, 1% of B-27 supplement 50X, 0.12% of GlutaMAX supplement, and 10 µg/mL of penicillin/streptomycin) 24 h later. The cells were kept in an incubator with 5% CO_2_, 37 °C, and controlled humidity until they reached 14 days in vitro (DIV 14). Cell culture media was changed every three days.

### 4.9. Neural Progenitor Cells Derived from Neurosphere (NPCdn) Culture

To analyze the ability of neural progenitor cells to spread and differentiate into mature neurons, spinal cord cells were harvested from neonates (males and females) of transgenic mice expressing green fluorescent protein (GFP) in P0–P3 (animal handling protocol approved by CEUA-UFABC 4509160816). As previously described, the animals were decapitated, and their spinal cords were dissected and maintained in cold buffer A. After sedimentation, cells were dissociated by incubation with 0.1% trypsin/EDTA for 15 min at 37 °C. FBS was added to stop trypsin action, and the remaining tissue was mechanically dissociated using 1 mL pipette tip. The cells were centrifuged for 5 min at 1500 rpm, and the supernatant was discarded, followed by resuspension in DMEM/F12-7 (ThermoFisher Scientific, Waltham, MA, USA), supplemented with 2% B27 (ThermoFisher Scientific, Waltham, MA, USA), 20 ng/mL of epidermal growth factor (EGF, Sigma St. Louis, MO, USA), 20 ng/mL of fibroblast growth factor (FGF2, R&D, Minneapolis, MN, USA), 1% penicillin/streptomycin (ThermoFisher Scientific, Waltham, MA, USA), and 5 μg/mL heparin (Sigma Aldrich, St. Louis, MO, USA). After mechanical dissociation, the cells were plated on a polyHEMA (Sigma Aldrich, St. Louis, MO, USA) precoated 75 cm^2^ flask at 2.4 × 10^7^ cells/flask or on a 24-well plate at a density of 2 × 10^6^ cells/well. Medium change was performed every 4–5 days by centrifugation for 5 min at 900 rpm, and half the volume of the medium was replaced by fresh medium [102]. Neurosphere formation took 14–21 days to reach 100 μm in diameter and be considered ready for experimental procedures [103].

### 4.10. Culture of Neural Progenitor Cells Derived from Human Induced Pluripotent Stem Cells (hiPSC-NPC)

To analyze the differentiation of human cells in direct contact with biomaterials, neural progenitor cells derived from human induced pluripotent stem cells (hiPSC-NPC) were used; these were cultured as previously described from the hiPSC line, 1-DL-01, from WiCell [104]. Experiments using hiPSC-NPC were conducted with the approval of the University of Victoria’s Human Ethics Committee under protocol number #12-187. Briefly, the cells were cultured in STEMdiff™ Neural progenitor medium (NPM) (05834, Stemcell Technologies, Vancouver, BC, Canada) containing 1X STEMdiff™ Neural Progenitor Supplement A (05836, Stemcell Technologies, Vancouver, BC, Canada), 1X STEMdiff™ Neural Progenitor Supplement B (05837, Stemcell Technologies, Vancouver, BC, Canada), and 1% penicillin-streptomycin (P4333, Sigma Aldrich, St. Louis, MO, USA). These medium supplements were used to promote the efficient conversion of hiPSC to CNS-type NPCs and inhibit the unwanted differentiation of non-CNS-type cells. The cultured hiPSC-NPC were maintained at 37 °C with 5% CO_2_, and media changes were performed daily.

### 4.11. Indirect Test and 3- [4,5-Dimethyl-Thiazol-2-Yl] -2,5-Diphenyltetrazolium Bromide (MTT) Assay

The indirect or extract test was performed according to ISO 10993-5:2009 [73]. For the assay, elution solutions of each biomaterial were prepared one day in advance, for which sterile pieces with an area of 3 cm^2^ for each 1 mL of medium were required. Vero cells were cultured for 24 h, while for SCC and NPCdn until DIV 7, the culture medium was replaced by the elution medium prepared for each biomaterial (CHI, PCL, and PLLA). The tests were carried out in triplicate with negative control (NEG) filter paper (known as noncytotoxic), and latex (known as cytotoxic), as a positive control (POS).

The MTT assay (Sigma Aldrich, St. Louis, MO, USA) evaluated cell viability by assessing mitochondrial metabolism. After 24 h of incubation, the elution medium was removed, the culture was washed twice with 100 µL of saline phosphate buffer (PBS) at 37 °C, and it was incubated with 100 µL of MTT solution 0.5 mg/mL in PBS for 4 h. After this time, the MTT solution was removed, and 50 µL of DMSO (dimethyl sulfoxide, Synth, Diadema, SP, Brazil) was added. The Elisa Reader (SpectraMax M5, Molecular Devices, San Jose, CA, USA) performed the plate reading at 570 nm. The control group (CTL), without biomaterial, was considered 100% viable cells and employed for normalization.

### 4.12. Direct Contact Test

The direct test was performed according to ISO 10993-5:2009 [73] to analyze the cell morphology of Vero cells, SCC, and NPCdn in direct contact with the biomaterials. Cells were incubated under experimental conditions until the contact test (Vero: DIV 1, SCC, and NPCdn: DIV 7). Biomaterials were gently placed above the cell layers, and 1 cm^2^ piece of each material and 1 mL of medium were used for each well (24-well plate). The previously mentioned POS and NEG used for the indirect test were also used in the direct test. The analysis was carried out under an inverted microscope, Nikon TS100F (Nikon Instruments Inc., Melville, NY, USA), after contact with the biomaterials for 24 h for Vero cells, DIV 7 for spinal cord cells, and DIV 7 for neurospheres. For better visualization, the neural cells were stained with cresyl violet (C5042, Sigma Aldrich, St. Louis, MO, USA).

### 4.13. Scanning Electron Microscopy (SEM)

After incubation, Vero: DIV 1; SCC and NPCdn: DIV 7, the cells were subjected to the fixation process using glutaraldehyde 2.5% diluted in PBS 0.1M and pH 7.4 for 1 h at room temperature. The samples were then washed and dehydrated in ethanol series (50%, 60%, 70%, 80%, and 90%) for 10 min each, and after that, two washes in absolute (100%) ethanol of 5 min each. To finalize the chemical dehydration process, a 100% solution of hexamethyldisilazane (HMDS) was used to immerse the samples overnight in an exhaustion hood for complete evaporation. At last, the stub was mounted with a double-sided sticky tape, and the samples were coated with a thin (15 nm) layer of gold by sputtering at room temperature (Leica ACE200, Leica Microsystems, Wetzlar, HE, Germany). After metallization, the samples were stored under vacuum until observed under SEM (Quanta 250TM, ThermoFisher Scientific, Waltham, MA, EUA). Images were taken under high vacuum at different magnifications with 5 kV.

### 4.14. Immunofluorescence Assay (IF)

Cells were fixed for 1h in 4% paraformaldehyde (PFA) in phosphate buffer (PB) 0.1 M at pH 7.3 and washed three times in PBS, followed by membrane permeabilization with 0.4% Triton X-100 diluted in PBS at 4 °C for 40 min. A 1% albumin in PBS at 4 °C for 40 min was used to block unspecific binding. After several washes with PBS, the cells were incubated overnight with primary antibodies, 1:500 βIII tubulin (TUJ1; ab78078, Abcam, Cambridge, UK)- a neuronal marker, and 1:500 glial fibrillary acidic protein (GFAP; G3893, Sigma Aldrich, St. Louis, MO, USA), a glial astrocytic marker, in a solution containing 5% normal donkey serum (D9663, Sigma Aldrich, St. Louis, MO, USA) and 0.3% Triton X-100 diluted in PB 0.1M at room temperature. After serial washes with PBS, the cells were incubated with 1:1000 Alexa 488 fluorescent secondary antibody (A11008, ThermoFisher Scientific, Waltham, MA, USA) diluted in 0.3% Triton X-100 on PB 0.1 M for 2 h at room temperature. The samples were counterstained with the nuclear marker 4′-6-diamino-2-phenylindole (DAPI). The same protocol was performed for double IF using 1:1000 Alexa 546 secondary antibody (A11018, ThermoFisher Scientific, Waltham, MA, USA). After washing, cells were imaged with Nikon TS100F inverted microscope (Nikon Instruments Inc., Melville, NY, USA).

### 4.15. Flow Cytometry

hiPSC-NPC was seeded on top of sterile scaffolds in 1 cm^2^ and incubated for DIV 15 under experimental conditions. The cells were then enzymatically dissociated from the biomaterials with 0.125% trypsin-EDTA and 25 mM of sodium citrate in PBS at 37 °C under the agitation of 25 rpm for 10 min. FBS was added for trypsin neutralization. The cells were washed three times by adding PBS and centrifuging at 600 g for 5 min. The cells were then fixed with flow cytometry fixation buffer (FC004, R&D Systems, Minneapolis, MN, USA) and incubated for 30 min at room temperature, then washed and resuspended in flow cytometry permeabilization/wash buffer I (1X) (FC005, R&D Systems, Minneapolis, MN, USA). Marker antibodies, TUJ1 (60052, Stemcell Technologies, Vancouver, BC, Canada) and GFAP (60048, Stemcell Technologies, Vancouver, BC, Canada), and isotype controls, mouse IgG2A PerCP-conjugated Isotype control (IC003C, R&D Systems, Minneapolis, MN, USA), were added per manufacturer’s instructions and incubated for 1 h at 4 °C in the dark. The cells were then washed twice with PBS, resuspended in washing buffer and the marker expression data was collected using a Guava EasyCyte HT flow cytometer (Millipore, Burlington, MA, USA).

### 4.16. Image and Statistical Analysis

Image analyses were performed with ImageJ software (v1.54f, National Institute of Mental Health, Bethesda, MD, USA), and data were exported to GraphPad Prism software version 6.0 (v8.0.1, GraphPad, San Diego, CA, USA) and Microsoft Excel (v2306, Microsoft, Redmond, WA, USA). One-way and two-way ANOVA followed by post hoc Tukey tests were used to compare more than two groups. The Student’s t-test was used to compare two groups. All results were presented as mean ± standard error of the mean. Statistical significance was set at a *p*-value of ≤0.05. Images and charts were prepared with Adobe Photoshop (v24.2.0, Adobe Systems Inc., San Jose, CA, USA). To address potential variability during fabrication, each analyzed sample (*n* value) is a single measure derived from a unique biomaterial sample. A minimum of three samples were utilized for each experiment to ensure robustness.

### 4.17. Quantification of Positive Cells in IF

Double-blinded manual counting was performed to determine the percentage of TUJ1- and GFAP-positive cells. TUJ1- and GFAP-positive cells were considered neurons and astrocytes, respectively. Data were shown as the percentage of all counted cells by image.

### 4.18. Quantification of Neurosphere Spread Ratio and Core Area

Using NeuronJ, a plugin for ImageJ, neurosphere cell outgrowth and core area were obtained from spheroids of GFP-NPCdn. The regions were separated between the cells protruding from the neurosphere and the cells composing the core area of each spheroid. These data were used to calculate the neurosphere spreading rate, a ratio between the cell outgrowth and neurosphere core areas [105].

### 4.19. Quantification of Soma Area, Number of Prolongations, and Median Prolongation Median Length

Using ImageJ, soma area, number, and length of cell prolongations were obtained from randomized cells. The median of prolongation length was calculated for each analyzed cell to obtained a representative value of this data. 3D plot and centroid data was obtained using Matlab (R2022b, Natick, MA, USA).

## 5. Conclusions

We evaluated and characterized the behavior of neural cells associated with polymeric biomaterials based on CHI, PCL, and PLLA. First, we verified the physical-chemical properties of the tested biomaterials and evaluated their cytotoxicity for different cell types (Vero, SCC, and NPCdn) by direct and indirect contact. According to our results, only CHI scaffolds stimulate inflammatory conditions, possibly due to their high charge density dictated by the degree of chitin deacetylation and molecular weight. These conditions may affect spinal cord neurons and progenitor cell behavior, impacting not only the viability of NPCdn, but also the attachment of SCC and NPCdn, as well as the expression of TUJ1 in SCC, and the neural differentiation of hiPSC-NPC. By contrast, PCL and PLLA, considered hydrophobic scaffolds, demonstrated moderate surface energy and allowed neuronal cells to attach and organize themselves in different populations.

Regarding hiPSCs, synthetic polymers promoted differentiation into neuronal phenotypes. These results disclose that CHI, despite its potential in neural tissue engineering, requires a deeper investigation of its specific features, such as degree of deacetylation, molecular weight, polydispersity, purity, form, and dosage, before considering its application in the CNS in in vivo trials. On the other hand, we concluded that PCL and PLLA scaffolds are promising for tissue engineering applications to develop spinal cord regeneration therapies.

## Data Availability

Not applicable.

## Supplementary Materials

The following supporting information can be downloaded at: https://www.mdpi.com/article/10.3390/ijms241713642/s1.
