# Supplementary material for: The Impact of Biomaterial Surface Properties on Engineering Neural Tissue for Spinal Cord Regeneration
Source: Int J Mol Sci. 2023 Sep 4;24(17):13642. doi: 10.3390/ijms241713642 (PMC10488158; doi:10.3390/ijms241713642)
Supplement: Supplementary file 1 [file ijms-24-13642-s001.zip › ijms-2574225-SI.pdf]

Supplementary Data:

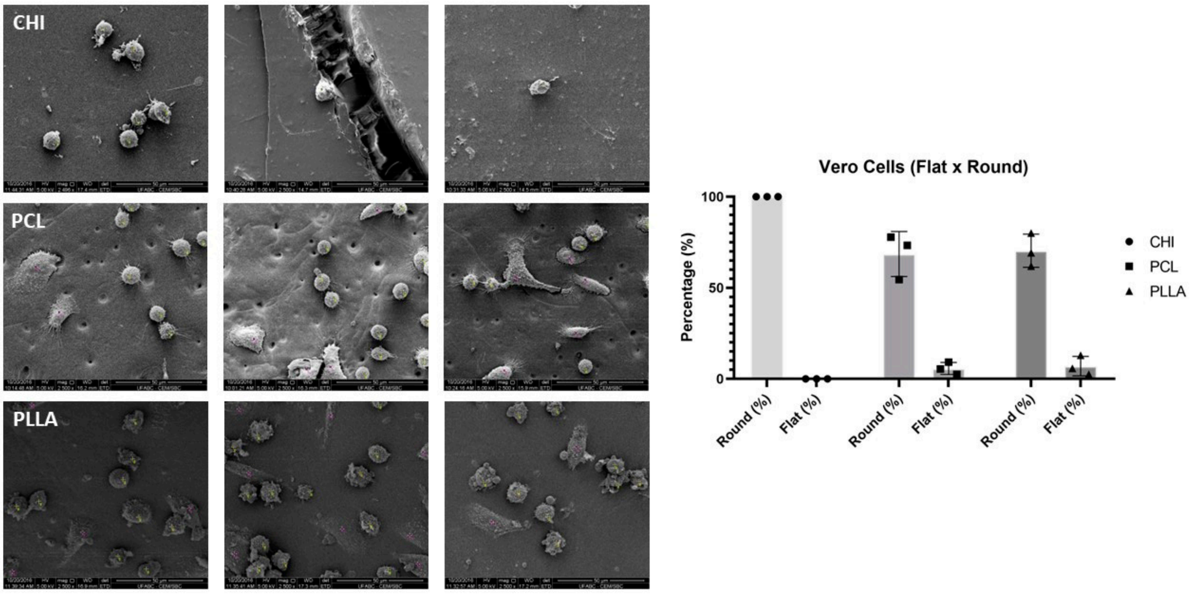

Supplementary Data S1. Comparison of round and flat VERO cells over scaffolds. Scanning Electron Microscopy Visualization of chitosan (CHI), poly (ε- caprolactone (PCL), and poly (L-lactic acid) (PLLA) scaffolds cultured with VERO cells. Comparison between flat and round cells. CHI exhibited an average of 100% round and 0% flat cells. PCL showed an average of 68.55% round and 5.73% flat cells. PLLA exhibited an average of 70.38% round and 7.07% flat cells.

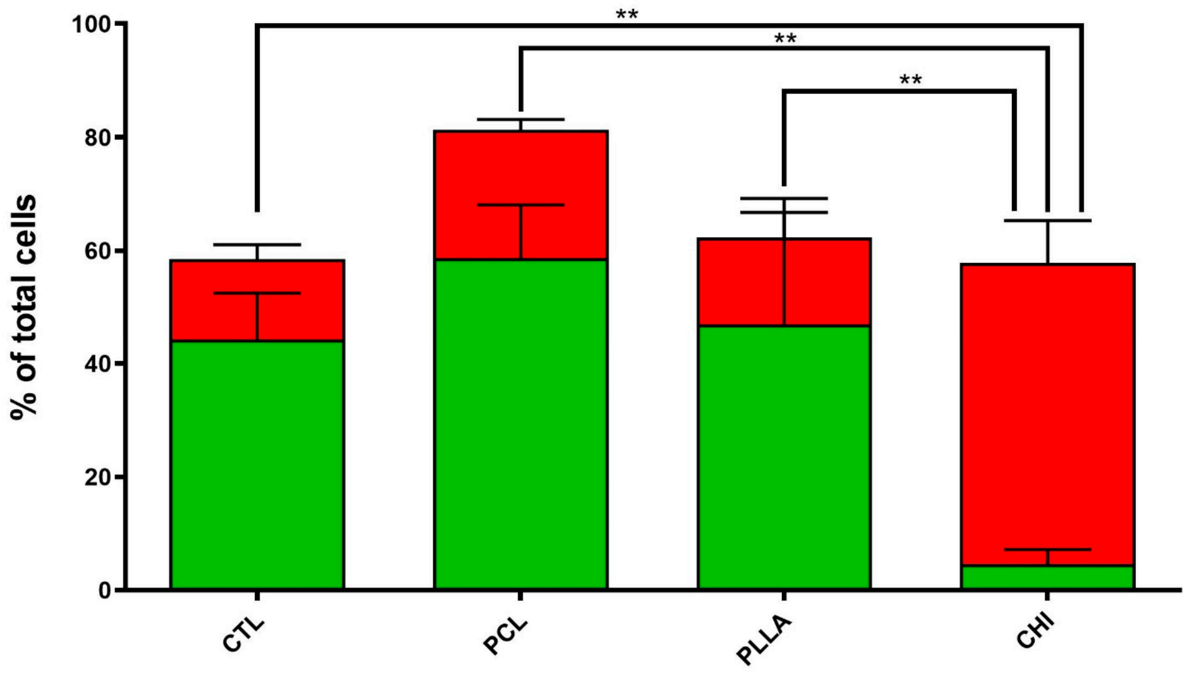

Supplementary Data S2. Comparison of cells 3D cultured over scaffolds to cells cultured in 2D. The emergence of βIII-tubulin (TUJ1) and glial fibrillary acidic protein (GFAP) in neural progenitor cells derived from human induced pluripotent stem cells (hiPSCs-NPC) cultivated over the scaffolds after DIV 15 in free specific growth factors conditions. Comparison of cells 3D cultured over poly (ε-caprolactone) (PCL), poly (L-lactic acid) (PLLA), and (D) chitosan (CHI) scaffolds to cells, 2D cultured in a petri dish (CTL). Green represents the percentage of TUJ1 and red represents the percentage of GFAP. Bars represent standard errors of the mean. ** P<0.05.
